# Supplementary material for: High prevalence of gastroschisis in Brazilian triple side border: A socioenvironmental spatial analysis
Source: PLoS One. 2021 Feb 26;16(2):e0247863. doi: 10.1371/journal.pone.0247863 (PMC7909679; doi:10.1371/journal.pone.0247863)
Supplement: Attachment — Submitted filename: PONE-D-20-30903 - Review 01.pdf [file pone.0247863.s002.pdf]

Dear Dr. JJ Cray

I am very thankful for the invitation to review the manuscript for PLOS ONE entitled "High incidence of gastroschisis in Brazilian triple side border: a socioenvironmental spatial analysis" (PONE-D-20-30903).

I did use the very best of my knowledge to help you decide and the authors to improve their manuscript.

Overall, the authors did an excellent research regarding the exposure of magnetic fields, and their association with live births with gastroschisis in Foz do Iguassu, a city located in Parana state – Brazil, which hosts one of the world's biggest hydroelectric dams.

They respected the manuscript organization present in the instruction to authors on the PLOS website. It has organized in Title, a non-structured Abstract, Background, Material and Methods, Results, Discussion, Limitation, Conclusion, Acknowledgments, References and presents the Result's figures at the end.

Observations regarding each manuscript section are below.

1) Title

It is specific, descriptive, and draws attention to the present question.

No recommendations in this section.

2) Abstract

It is a non-structured abstract that describes the study's primary objective, explains the method's principal points, and shows the main results and conclusion.

No recommendations in this section.

3) Background

The authors summarized the gastroschisis problem very well; nevertheless, PREVALENCE is the usual term in the medical literature when referring to a frequency measure of any congenital disease, not INCIDENCE.[1, 2]

I recommend altering the term INCIDENCE for PREVALENCE in this and all the other sections and figures.

1. Hook EB. Incidence and prevalence as measures of the frequency of birth defects. American journal of epidemiology. 1982;116(5):743-7.
2. Mason CA, Kirby RS, Sever LE, Langlois PH. Prevalence is the preferred measure of frequency of birth defects. Birth defects research Part A, Clinical and molecular teratology. 2005;73(10):690-2. Epub 2005/10/22. doi: 10.1002/bdra.20211. PubMed PMID: 16240384.

4) Materials and Methods

A) Study design, setting, and population

Line 74 - The term newborn covers the population of live births and stillbirths. As SINASC uses only the live births population, it would be better to replace the term to avoid confusion.

Line 74 – Is not the six years period (2012 – 2017) short for this analysis? Publications numbers 27 – 30 in the REFERENCES section present a study period that varies from 7 to 33 years.

The alteration of the term and the explanation for the six-year period are the recommendations in this section.

B) Data sources and study variables

All the SINASC database is available on the DATASUS website (www.datasus.gov.br), including the MICRODATA used in this research (http://www2.datasus.gov.br/DATASUS/index.php?area=0901&item=1&acao=28&pad=31655).

My recommendation in this section is to:

- Alter the statement in "Data Availability": NO – to YES, and refer to the DATASUS WEBSITE.
- Alter the statement that "Data cannot be shared publicly" because they already are public. In "Describe where the data may be found……appropriate details."
- The technical details should be expanded and clarified to ensure that readers understand precisely the steps made. It is not only to export the SINASC data to an Excel spreadsheet; TABWIN should be used first.
- The technical details should be expanded and clarified to ensure that readers precisely understand the steps to obtain all the SINASC and IBGE data.

C) Power Transmission Lines

No recommendations in this section.

D) Data analysis

The alteration to PREVALENCE instead of the term INCIDENCE as recommended before.

E) Global Moran's Index

No recommendations in this section.

F) Local indicator of spatial association analysis

No recommendations in this section.

G) Getis-Ord statistics

No recommendations in this section.

H) Spatial scan statistic

No recommendations in this section.

I) Logistic regression

Line 208: The authors used a distance of 850 meters between the centroid of the census sector to the closest point in PTL as an independent variable. No other paper in the REFERENCE section uses this distance (Ref 10: 600m, Ref 29: <200m, 200-600m, >600m, Ref 30: 500m), and in the DISCUSSION section line 272, the authors also describes the distances. So, the question is, why did the authors use 850m as standard?

The authors should clarify and expand the technical details to a better understanding.

Line 220: The authors could create another subsection entitle: Ethics Review to present the Ethics data from Plataforma Brasil, apart from Logistic regression.

5) Results

Line 225: (15 / 26,182) X 10,000 = **5.73 (PREVALENCE** rate 2012 - 2017) not 5.75

Line 266, 228 & 241: Change the term Incidence for Prevalence

FIG 1, 2, 3 & Table 1: Change the term Incidence for Prevalence

6) Discussion

While the study appears to be sound, the language is unclear, making it difficult to follow. Please advise the authors to work with a writing coach or copyeditor to improve the text's flow and readability, principally in the Discussion's first paragraph.

7) Limitation

No recommendations in this section.

8) Conclusion

No recommendations in this section.

9) Acknowledgments

No recommendations in this section.

10) References

No recommendations in this section.

Overall, the manuscript's idea is outstanding. A major revision will be required, and an English revision from a Native speaker or a writing editing service. Some points must be better explained to clarify and give a better understanding to the readers.

Sincerely

M.G.C - M.D, Ph.D
