# Supplementary material for: High prevalence of gastroschisis in Brazilian triple side border: A socioenvironmental spatial analysis
Source: PLoS One. 2021 Feb 26;16(2):e0247863. doi: 10.1371/journal.pone.0247863 (PMC7909679; doi:10.1371/journal.pone.0247863)
Supplement: Attachment — Submitted filename: Response to Reviewers.docx [file pone.0247863.s003.docx]

**RESPONSES TO THE REVIEWERS**

PONE-D-20-30903

High incidence of gastroschisis in Brazilian triple side border: a socioenvironmental spatial analysis

Dear Reviewers,

We would like to thank you for the careful review and contribution to the paper. Journal requirements and reviewers´ comments are addressed in the responses bellow. Also, a revised marked version is also presented with changes highlighted in yellow.

Yours sincerely,

Suzana de Souza

**Journal Requirements:**

*2. In the methods section, please provide additional information regarding how study variables were extracted from the database for analysis. Please ensure that you have described this in sufficient detail to allow your work to be replicated.*

Authors’ answer: We have provided more information regarding study variables. Please see line 79 in *Data sources and study variables* subsection*:*

“The variables obtained from SINASC were:

• Type of congenital anomaly (Gastroschisis (Q79.3, according to International Classification of Diseases – ICD));

• Parturients age (Presented at SINASC as a continuous quantitative variable; in this research was categorized as adolescent (up to 19 years old), adult (20 to 34 years old) and advanced age (over 35 years old)).

• Prenatal start period (Presented at SINASC as a continuous quantitative variable; in this research was categorized as early prenatal care (beginning in the first semester of pregnancy) and late prenatal care (beginning after the first semester of pregnancy)).”

*3. We note that you have indicated that data from this study are available upon request. PLOS only allows data to be available upon request if there are legal or ethical restrictions on sharing data publicly.*

**Authors’ answer:** Data set are not subject to legal or ethical restriction. Minimal anonymized was upload to protect patient information.

4. *We note that [Figure(s) 2, 3 and 4] in your submission contain map images which may be copyrighted. All PLOS content is published under the Creative Commons Attribution License (CC BY 4.0), which means that the manuscript, images, and Supporting Information files will be freely available online, and any third party is permitted to access, download, copy, distribute, and use these materials in any way, even commercially, with proper attribution. For these reasons, we cannot publish previously copyrighted maps or satellite images created using proprietary data*.

**Authors’ answer:** Brazilian Institute of Geography and Statistics (IBGE) declared all extracted data (shapefile) is public and can be freely reproduced with source indicated. We have indicate source in each image. Written permission is upload. Please also see lines 230, 236 and 243 in *Results section*: “Source: Brazilian Institute of Geography and Statistics, 2010. SIRGAS2000 projection / UTM zone 21S.”

**Reviewers' comments:**

*3) Background*

*The authors summarized the gastroschisis problem very well; nevertheless, PREVALENCE is the usual term in the medical literature when referring to a frequency measure of any congenital disease, not INCIDENCE.*

*I recommend altering the term INCIDENCE for PREVALENCE in this and all the other sections and figures.*

**Authors’ answer:** We have changed the term INCIDENCE to PREVALENCE in these passages. Please see lines 44, 122, 219 and 272.

4) Materials and Methods

*A) Study design, setting, and population*

*Line 74 - The term newborn covers the population of live births and stillbirths. As SINASC uses only the live births population, it would be better to replace the term to avoid confusion.*

**Authors’ answer:** We have now used only the term "live births".

*Line 74 – Is not the six years period (2012 – 2017) short for this analysis? Publications numbers 27 – 30 in the REFERENCES section present a study period that varies from 7 to 33 years.*

*The alteration of the term and the explanation for the six-year period are the recommendations in this section.*

**Authors’ answer:** Live Birth Information System (SINASC) was created in 1990 but its implementation occurred only gradually in all Federation Units. We chose this period to collect more recent and consistent data to minimize bias in the study.

*D) Data analysis*

*The alteration to PREVALENCE instead of the term INCIDENCE as recommended before.*

**Authors’ answer:** We have changed the term INCIDENCE to PREVALENCE in these passages.

*I) Logistic regression*

*Line 208: The authors used a distance of 850 meters between the centroid of the census sector to the closest point in PTL as an independent variable. No other paper in the REFERENCE section uses this distance (Ref 10: 600m, Ref 29: <200m, 200-600m, >600m, Ref 30: 500m), and in the DISCUSSION section line 272, the authors also describes the distances. So, the question is, why did the authors use 850m as standard?*

*The authors should clarify and expand the technical details to a better understanding.*

**Authors’ answer:**, Unlike other studies, our distance analysis was based on the centroid of each census sector. After empirical tests, we found that the best distance to cover all census sectors near the power transmission lines was 850 meters. We added a sentence in order to better explain this choice. Please see line 202 in *Logistic regression* subsection: “After empirical tests, the distance that included all census sector close to PTL was 850 meters”.

*Line 220: The authors could create another subsection entitle: Ethics Review to present the Ethics data from Plataforma Brasil, apart from Logistic regression.*

**Authors’ answer:** We created a new subsection according to the suggestion. Please see line 213.

*5) Results*

*Line 225: (15 / 26,182) X 10,000 = 5.73 (PREVALENCE rate 2012 - 2017) not 5.75*

**Authors’ answer:** We fixed the error in the sentence.

*Line 266, 228 & 241: Change the term Incidence for Prevalence*

*FIG 1, 2, 3 & Table 1: Change the term Incidence for Prevalence*

**Authors’ answer:** We have changed the term INCIDENCE to PREVALENCE in all sections of the manuscript.

*6) Discussion*

*While the study appears to be sound, the language is unclear, making it difficult to follow. Please advise the authors to work with a writing coach or copyeditor to improve the text's flow and readability, principally in the Discussion's first paragraph.*

**Authors’ answer:** The manuscript was extensively revised. A new version is presented after English grammar and language improvements.
